# Discrimination between human and animal blood by attenuated total reflection Fourier transform-infrared spectroscopy

**DOI:** 10.1038/s42004-020-00424-8

**Published:** 2020-12-10

**Authors:** Ewelina Mistek-Morabito, Igor K. Lednev

**Affiliations:** Department of Chemistry, University at Albany, State University of New York, 1400 Washington Avenue, Albany, NY 12222 USA

**Keywords:** Bioanalytical chemistry, Infrared spectroscopy

## Abstract

Forensic chemistry is an important area of analytical chemistry. This field has been rapidly growing over the last several decades. Confirmation of the human origins of bloodstains is important in practical forensics. Current serological blood tests are destructive and often provide false positive results. Here, we report on the development of a nondestructive method that could potentially be applied at the scene for differentiation of human and animal blood using attenuated total reflection Fourier transform-infrared (ATR FT-IR) spectroscopy and statistical analysis. The following species were used to build statistical models for binary human–animal blood differentiation: cat, dog, rabbit, horse, cow, pig, opossum, and raccoon. Three other species (deer, elk, and ferret) were used for external validation. A partial least squares discriminant analysis (PLSDA) was used for classification purposes and showed excellent performance in internal cross-validation (CV). The method was externally validated first using blood samples from new donors of species used in the training data set, and second using donors of new species that were not used to construct the model. Both validations showed excellent results demonstrating potential of the developed approach for nondestructive, rapid, and statistically confident discrimination between human and animal blood for forensic purposes.

## Abstract (web summary)

Statistical models applied to spectroscopic data offer a promising alternative to lab-based assays for forensic analysis. Here human blood is distinguished from that of 11 animal species using attenuated total reflection Fourier transform-infrared spectroscopic data.

## Introduction

Analytical chemistry plays an important role in forensic science. Locard’s exchange principle says that “when two objects come into contact, material between them can be transferred”^1^. It is the role of analytical chemistry to detect and identify this material. During the last decade, a variety of emerging analytical and bioanalytical methods have been developed for forensic serology, toxicology, and trace analysis^1–3^. Another significant development in forensic analysis is the introduction of advanced statistics that increase the reliability of the results, reduce the “human factor”, and provide information about the confidence interval, which is important for consideration of evidence in court^1,2,4^.

Bloodstains found at crime scenes are very important evidence and one of the main sources of DNA. Identification of blood can involve several analytical steps^5^. An example protocol for the examination of blood involves physical examination of evidence, recording of evidence, preliminary tests, and confirmatory tests. Preliminary tests are also known as presumptive or screening tests and can narrow the search field. These tests are based on reactions between blood components and a specific reagent. However, there are other body fluids and substances that can react positively to these tests. Confirmatory tests, which also use reactions between blood components and reagents, are more specific for identification of blood. The two most common confirmatory assays for blood are the Teichmann and Takayama tests, which are both microcrystal tests^5^. Immunoassay tests, such as ABAcard® HemaTrace® and HemDirect Hemoglobin are routine human blood identification methods^6,7^. These tests are based on the reaction with hemoglobin and are specifically sensitive to human and higher primate bloods. Other species (e.g., ferret) can cause false positive results with some of these tests because of the hemoglobin composition, which is similar to that of human^7,8^. Although some confirmatory tests can be conducted at a crime scene, the others require laboratory settings. Both presumptive and confirmatory assays are destructive in nature. The National Institute of Justice recently recommended that laboratories should consider going directly to DNA examination and perform serology only if necessary^9^. Discrimination between human and animal blood is especially important for hit-and-run cases when a suspect claims an incident involved an animal and not a human. In such cases, it is very advantageous to test any bloodstains on the suspect’s car to determine their origin.

De Wael et al.^10^ was the first to report on the use of vibrational spectroscopy for discrimination between human and animal blood. They concluded that it was impossible to visually differentiate spectra of blood from different species. Our laboratory has demonstrated that Raman spectroscopy in combination with chemometrics allows for identification of species using bloodstain analysis^8,11–13^. Bai et al.^14^ also applied Raman spectroscopy and advanced statistical analysis to differentiate between human and animal blood. Fujihara et al.^15^ demonstrated that a portable Raman spectrometer could be successfully used for this purpose. Diffuse reflectance spectroscopy and spatially resolved near-infrared transmission spectroscopy were employed for differentiating human and animal blood^16–20^.

Attenuated total reflection Fourier transform-infrared (ATR FT-IR) spectroscopy is a vibrational spectroscopic technique that has a wide range of applications in forensics^1,3^. This technique performs well for the examination of body fluid traces, including the blood^21,22^. In an earlier study, we showed that ATR FT-IR spectroscopy in combination with advanced statistics could differentiate between human, cat, and dog bloodstains^23^. Lin et al.^24^ used ATR FT-IR spectroscopy to examine blood from a wider range of species and bloodstains subjected to various indoor and outdoor conditions.

Here, we expanded upon species identification work using ATR FT-IR spectroscopy and statistical analysis. The data set consisted of blood spectra from human samples and eight animal species that were all mammals (cat, dog, rabbit, horse, cow, pig, opossum, and raccoon). Three different species (deer, elk, and ferret) were used for external validation. The pool of species was chosen to include animals that are common as pets, consumed by humans, or can be involved in wildlife crimes or hit-and-run accidents. ATR FT-IR spectroscopy was used to collect spectra of blood samples from the different species. A partial least squares discriminant analysis (PLSDA) was used to distinguish human blood from animal blood. PLSDA is a supervised modeling technique that uses a calibration data set to train the model and allows for predictions with an external validation set. In addition, a genetic algorithm (GA) was used to select the most informative variables (spectral regions) for differentiating between ATR FT-IR spectra of human and animal blood. This step was performed to gain an understanding of the statistically important parts of the blood spectrum and biochemical changes using characteristic bands.

## Results and discussion

In this study, ATR FT-IR spectroscopy was combined with advanced statistical analysis to distinguish human blood from that of a range of animal species. This study is a continuation of previously published work where differentiation between human, cat, and dog blood samples was conducted^23^. To extend on the previously reported work, we expanded the library to include additional species (rabbit, horse, cow, pig, opossum, and raccoon) and added external species (deer, elk, and ferret) for predictions.

### Spectral analysis

**Fig. 1:**
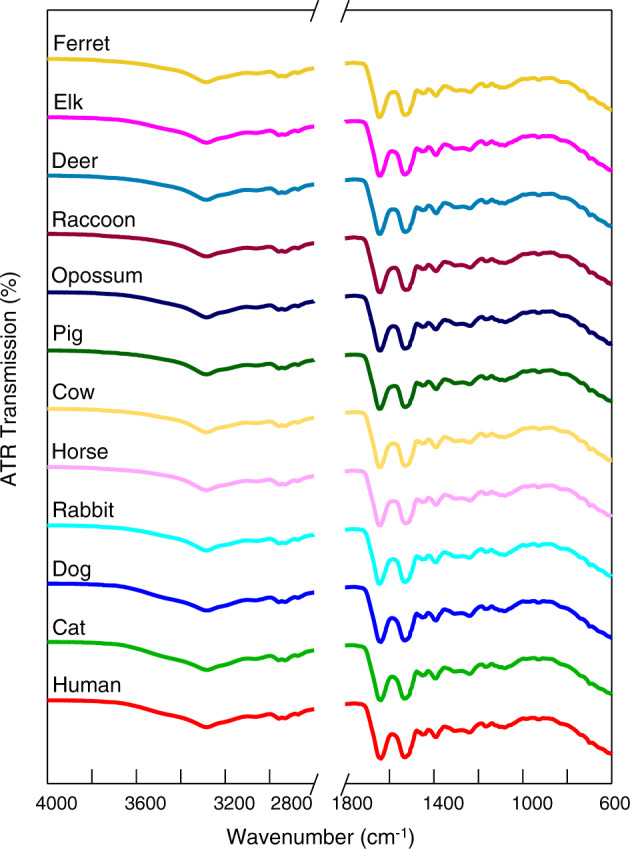
The averaged raw ATR FT-IR spectra of blood for different species. Spectral ranges: 4000–2800 and 1800–600 cm^−1^.

The ATR FT-IR spectra of blood (Fig. 1) obtained from different species were very similar and contained the same number of bands at the same positions. The spectral ranges of 4000–2800 and 1800–600 cm^−1^ were used, as they showed contributions in the biochemical compositions of the biological samples^25,26^. The following characteristic FT-IR spectral features were observed for the biological samples: lipids (3000–2800 cm^−1^), proteins (1700–1500 cm^−1^), nucleic acids (1250–1000 cm^−1^), and carbohydrates (1000–800 cm^−1^)^27,28^. Table 1 contains the representative band assignments of molecular vibrations for the FT-IR spectra of blood. As differentiation between spectra by visual inspection was impossible, advanced statistical analysis was used.

**Table 1:** Assignment of the infrared bands in human blood.

Wavenumber (cm^−1^)	Assignment
3500–3200	Water and hydroxyl (O–H stretching)^27,37^
3284	Amide A (N–H stretching)^25,38,39^
2958	Lipids (asymmetric stretching of CH_3_)^25,27,37,39^
2872	Lipids (symmetric stretching of CH_3_)^39^
1700–1600	Amide I (C=O stretching)^25,27,37–39^
1560–1500	Amide II (N–H bending and C–N stretching)^25,27,37–39^
1390	Lipids and proteins (symmetric bending of CH_3_)^25,37^
1239	Amide III (C–N stretching)^25,37,38^
1082	Glucose (C–O stretching)^25,39^
698	Amide IV (C–H bending)^39^

**Fig. 2:**
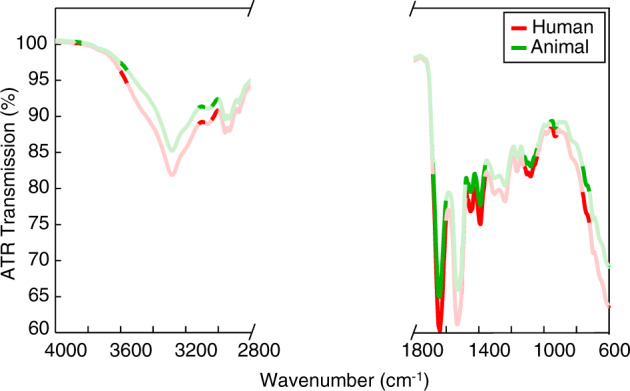
The averaged raw ATR FT-IR spectra of human blood (red) and animal blood (green). Featured spectral regions were selected by a genetic algorithm.

The GA was used to select the most informative spectral regions (Fig. 2 and Supplementary Table 1) for differentiating between the ATR FT-IR spectra of human and animal blood. Within the selected regions, the largest contribution to the differentiation was from proteins, mostly amide I at 1650 cm^−1^, lipids and proteins at 1390 cm^−1^, nucleic acids, and carbohydrates, such as glucose at 1082 cm^−1^.

Unfortunately, differences in blood composition between different species are not well known. Some of the studied species have different concentrations of hemoglobin, carbohydrates (mostly glucose), various hormones, and enzymes^29^. Also, blood types within different species vary^30^. Red blood cell morphometry, including the diameters, circumferences, and surfaces of erythrocytes, also varies among different species^31^. However, no specific dissimilarities between the studied species were found.

### Human–animal blood differentiation using ATR FT-IR spectroscopy

**Fig. 3:**
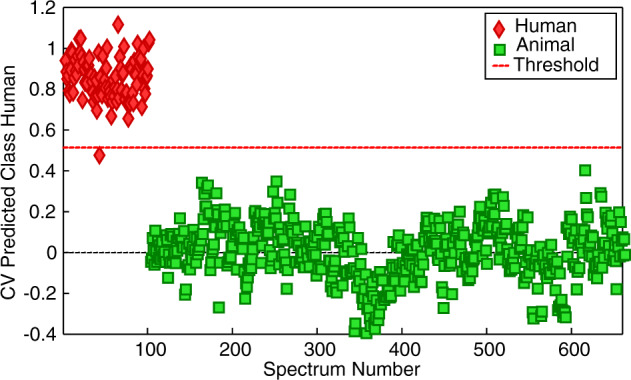
Cross-validated prediction results for the human class using the human–animal PLSDA model with six latent variables from blood samples. Each symbol corresponds to an individual ATR FT-IR spectrum with 10 spectra per donor. Human blood spectra are shown as red diamonds and animal blood spectra are shown as green squares. The red dotted line is the threshold for classification of spectra in the human class. Only one spectrum from a human sample is misclassified.

After the spectra were preprocessed, a binary PLSDA model using six latent variables (LVs) was built to classify the human and animal ATR FT-IR spectra of blood. The prediction results of cross-validation (CV) for samples in the human class can be found in Fig. 3. The human class was assigned a value of 1 and all human blood spectra should be located close to 1 on the plot and above the threshold (red dotted line). Spectra in the animal class should be located below the threshold and close to 0 on the plot. The CV prediction plot showed only one human blood spectrum misclassified in the animal class. The donor-level predictions indicated no misclassifications. When a second model was built using five LVs, the CV prediction plot illustrated complete separation between the human and animal blood ATR FT-IR spectra and no misclassifications were observed (Supplementary Fig. 1). The model showed 100% accuracy for differentiating between human and animal classes using the ATR FT-IR spectra of blood.

### External validation for human–animal discrimination

**Fig. 4:**
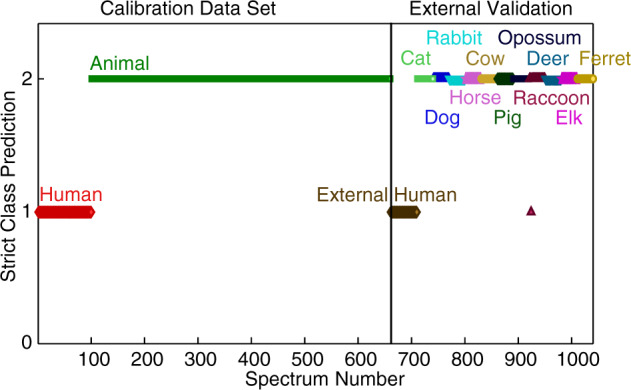
Strict class prediction results by the human–animal PLSDA model with six latent variables for blood samples. Human blood spectra (red diamonds) are assigned a value of 1 and animal blood spectra (green squares) are assigned a value of 2. All spectra of external samples are on the right side of the plot (spectra numbers 661–1040).

Once the PLSDA models were created and showed good internal prediction abilities, they were externally validated using new donors of the same species used in the calibration set, and external species that were not included in the training set. The strict class prediction plot for external samples using the PLSDA model with six LVs is shown in Fig. 4. The human class was assigned a value of 1, which meant that all human blood spectra should be aligned with 1 on the plot. The animal class was assigned a value of 2, which meant that all animal blood spectra should be aligned with 2 on the plot. Spectra of external predictions are shown per species (multiple species in the animal class). From the prediction results of the human–animal PLSDA model with six LVs, all 50 human blood spectra were correctly classified in the external validation. Looking at the external validation of the animal class with species used in the training data set, 239 out of 240 animal blood spectra were correctly classified. One spectrum that was misclassified in the human class was from a raccoon. However, when predictions were made at the donor level, all animal samples were correctly predicted. The results of external validations are given in Table 2. Prediction results for the second PLSDA model with five LVs are shown in Supplementary Fig. 2 and Supplementary Table 2. According to the prediction results of the human–animal PLSDA model with five LVs, 49 out of 50 human blood spectra were correctly classified in the external validation. One spectrum of an external human sample was misclassified. However, when classifications were made at the donor level, all human samples were correctly classified. Looking at the external validation of the animal class with species used in the training data set, 237 out of 240 animal blood spectra were correctly classified. Three spectra that actually came from raccoons were misclassified in the human class. However, when classifications were made at the donor level, all animal samples were correctly classified.

**Table 2:** Prediction results of external samples by the human–animal PLSDA model using six latent variables.

	Predicted as human (spectral level)	Predicted as animal (spectral level)	Donor-level prediction
Human	50	0	5/5
Cat	0	30	3/3
Dog	0	30	3/3
Rabbit	0	30	3/3
Horse	0	30	3/3
Cow	0	30	3/3
Pig	0	30	3/3
Opossum	0	30	3/3
Raccoon	1	29	3/3
Deer	0	30	3/3
Elk	0	30	3/3
Ferret	0	30	3/3

The table shows results at the spectral level and the donor level (when more than 50% of spectra from one donor are correctly classified).

In addition, three external species from outside of the training data set, with three donors each, were used for predictions with both PLSDA models. None of the blood spectra belonging to deer, elk, or ferrets were classified as human samples (Fig. 4 and Supplementary Fig. 2). These samples of new species were not unassigned because the biochemical differences between species are still not well known. However, the most significant point for forensic purposes is that none of the spectra were assigned to the human class. The results of external validations are given in Table 2 and Supplementary Table 2. It is very important to note here that ferret blood can result in false positive identification as human blood using some of the current forensic tests for human blood identification^8^. Our method showed no false positive results for this species. These results show this approach has excellent ability to discriminate between human and animal samples using ATR FT-IR spectra of blood.

The two models constructed here showed excellent results for both internal and external validation methods. While model with five LVs showed perfect internal CV results (100% sensitivity and specificity), it resulted in four misclassified spectra, including one human blood spectrum, from the total of 290 spectra for the first part of external validation, when new samples were used. When the model was built using six LVs, the internal CV results showed one misclassified blood spectrum from the total of 660 spectra. However, only a single animal blood spectrum was misclassified from the total of 290 spectra used in the first part of external validation. Most importantly, all spectra from external human blood samples were predicted correctly. In addition, both models showed no misclassifications of external animal species from outside of the training data set to human class.

Because of the outstanding performance of this method and the nondestructive nature, this technique is promising for application in practical forensics for human blood identification. Another advantage of this technique is the potential for in situ analysis using portable instruments. These aspects make this technique promising for forensic examination of body fluid traces at crime scenes. In this study, dry blood was scraped from the glass slide and deposited onto an ATR crystal for analysis. This allowed us to eliminate glass substrate interference. Similar approach can be used for bloodstains on other substrates provided that a sufficient amount of blood is present. In addition, we plan to work on an alternative approach in the future, which will involve direct ATR FT-IR measurements from bloodstain traces on various substrates. Commercial ATR FT-IR spectrometers, which allow for such direct measurements, are available. In addition, similar spectral characteristics of bloodstains could be acquired using other spectroscopic techniques, including diffuse reflection infrared spectroscopy and external reflection FT-IR spectroscopy^32,33^. However, a signal from the substrate contributes to the measured spectra and it needs to be taken into consideration. A similar problem has been tackled while Raman spectroscopy was used for the identification of biological stains on various interfering substrates^34,35^. Most recently, a multivariate statistical approach has been developed for detecting trace amounts of semen on strongly interfering substrates using Raman spectroscopy^36^.

## Conclusions

ATR FT-IR spectroscopy in combination with chemometrics shows excellent potential for nondestructive and rapid identification of human blood for forensic purposes. Human, cat, dog, rabbit, horse, cow, pig, opossum, and raccoon blood samples were used to build binary human–animal classification model. Ten spectra were measured for each individual blood sample. The constructed model with six LVs showed only one misclassification of a human blood spectrum in the animal class using internal CV. The method was externally validated first using blood samples from new donors of species used in the training data set, and second using donors of new species that were not used to construct the model. In the first case, the results of strict class prediction for a PLSDA model with six LVs showed only one misclassified spectrum out of 290 spectra (99.6% accuracy). However, when looking at the prediction results at the donor level, 100% accuracy was achieved and all samples were correctly classified. In the second case, none of the external species blood spectra (deer, elk, ferret) were misidentified as human. These results demonstrate the excellent performance of the method and its potential for application to a wide range of different species.

The major advantage of the presented approach is the nondestructive nature of ATR FT-IR spectroscopic measurements and the potential for in situ analysis. Before this technique can be applied to practical forensic investigations, further research is required to address some obstacles that are associated with crime scenes. First, most evidence found at crime scenes is deposited on different substrates. Therefore, studies of body fluid stains on various substrates need to be performed. Moreover, contaminated, aged, and diluted samples should be examined using ATR FT-IR spectroscopy. Once fully developed, this approach utilizing ATR FT-IR spectroscopy with advanced statistical analysis will be a promising method for analyzing body fluids at the crime scene.

## Methods

### Blood samples

Blood samples used in this study were purchased from BioIVT (Westbury, NY). In total, 15 human blood samples from subjects of different sexes, races, and ages, and 89 animal blood samples were used for differentiating between human and animal blood. The following species were used for comparison with the human samples: cat, dog, rabbit, horse, cow, pig, opossum, raccoon, deer, elk, and ferret. All samples were kept frozen until required for analysis. After defrosting and vortex mixing each sample, a 30-μL drop of blood was deposited onto a microscope slide and left to dry overnight. A sample of the dry blood was scraped from the glass slide onto an ATR crystal for analysis.

### Instrumentation and settings

Spectral measurements were performed using a PerkinElmer Spectrum 100 FT-IR spectrometer (PerkinElmer, Inc., Waltham, MA) with a diamond/ZnSe ATR crystal and PerkinElmer, Inc. Spectrum software version 6.0.2.0025 (PerkinElmer, Inc., Waltham, MA). Spectra were collected in the range of 4000–600 cm^−1^ with a resolution of 4 cm^−1^. A background check was performed before each sample was placed on the crystal. Before analysis of each sample, the crystal was cleaned with deionized water and acetone. After analysis, the sample was covered with 10% bleach for 15 min to disinfect it. Ten spectra were collected from various spots on each sample and multiple scans were performed for each spectrum (ten for human, cat, and dog samples, and three for rabbit, horse, cow, pig, opossum, raccoon, deer, elk, and ferret samples).

### Data analysis

Data treatment and statistical analysis were performed in PLS Toolbox 8.5.2 (Eigenvector Research, Inc., Wenatchee, WA) operating in Matlab R2017b version 9.3.0.713579 (MathWorks, Inc., Natick, MA). The samples were divided into training (calibration) and external validation data sets. The following species were used for the calibration set: human, cat, dog, rabbit, horse, cow, pig, opossum, and raccoon. The training data set consisted of 10 human blood samples and 56 animal blood samples (seven samples per species). The validation data set contained a minimum of 30% of the samples from each class: five human samples and three samples from each animal group. Additional validation was performed using external species (deer, elk, and ferret) from outside the training data set. Spectra were truncated to 4000–2800 and 1800–600 cm^−1^ regions. The following preprocessing steps were applied: transformation of the transmission to absorbance (log(1/*T*)), first-order derivative with a second polynomial, normalization by total area, and mean centering. A GA was performed to select the most informative spectral regions for differentiating between ATR FT-IR spectra of human and animal blood. The population size was 72 and generations number was 100. Double crossover was set for the breeding crossover rule, and the mutation rate was 0.005. The final data set selected by GA was a result of 200 runs. Two binary PLSDA classification models were built for differentiating between ATR FT-IR spectra of human and animal blood: (i) using five and (ii) six LVs. These numbers of LVs were chosen based on the local minima of classification error. The performance of these models was tested using internal and external validation. Both models were created using spectral ranges of 4000–2800 and 1800–600 cm^−1^. PLSDA is a supervised type of modeling that uses data for training the model. Internal validation was performed using the venetian blinds method of CV with ten splits. The CV process divides the data set so that some subsets are used for training the model and some are used for validation. The procedure is continued until all spectra are used for validation.

### Reporting summary

Further information on research design is available in the Nature Research Reporting Summary linked to this article.

## Supplementary information

Supplementary Information

Reporting Summary

## Data Availability

The data sets generated during and/or analyzed during the current study are available from the corresponding author on reasonable request.
