# Supplementary material for: Discrimination between human and animal blood by attenuated total reflection Fourier transform-infrared spectroscopy
Source: Commun Chem. 2020 Dec 10;3:178. doi: 10.1038/s42004-020-00424-8 (PMC9814708; doi:10.1038/s42004-020-00424-8)
Supplement: Supplementary file 1 — Supplementary Information [file 42004_2020_424_MOESM1_ESM.pdf]

SUPPLEMENTARY INFORMATION

Discrimination between human and animal blood for by attenuated total reflection Fourier transform-infrared spectroscopy

Ewelina Mistek-Morabito and Igor K. Lednev*

Department of Chemistry, University at Albany, State University of New York, 1400 Washington Avenue, Albany, NY 12222, United States

*Corresponding author. Tel: +1 518 591 8863, e-mail: ilednev@albany.edu (I.K. Lednev).

Supplementary Table 1. Spectral regions of ATR FT-IR spectra of blood selected by a genetic algorithm for data set for differentiation between human and animal.

**Selected wavenumbers by GA for human-animal discrimination (cm^{-1})**
3880-3841
3600-3561
3120-3081
3040-3001
1681-1602
1481-1362
1121-1042
961-922
761-722
601-600

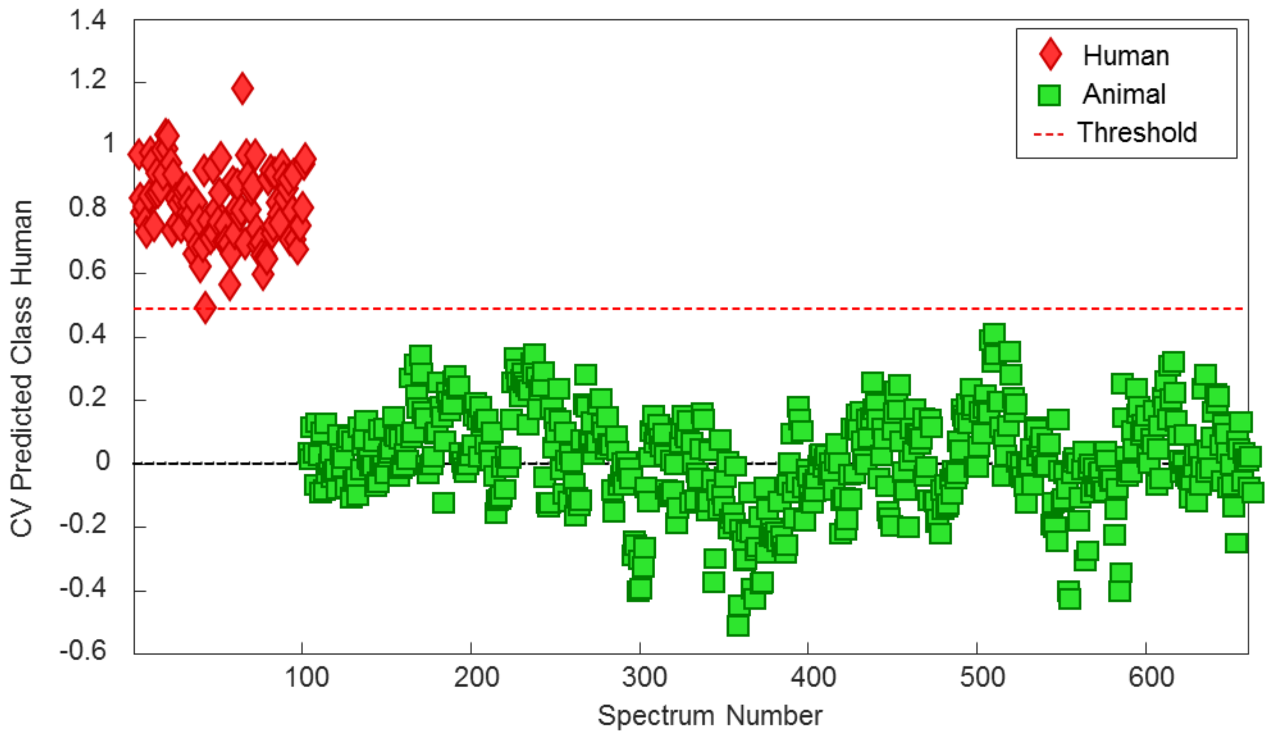

Supplementary Fig. 1 Cross-validated prediction results for the human class using the human–animal PLSDA model with five latent variables from blood samples. Each symbol corresponds to an individual ATR FT-IR spectrum with 10 spectra per donor. Human blood spectra are shown as red diamonds and animal blood spectra are shown as green squares. The red dotted line is the threshold for classification of spectra in the human class. The perfect classification of all spectra included in the training data set for both human and animal classes is achieved.

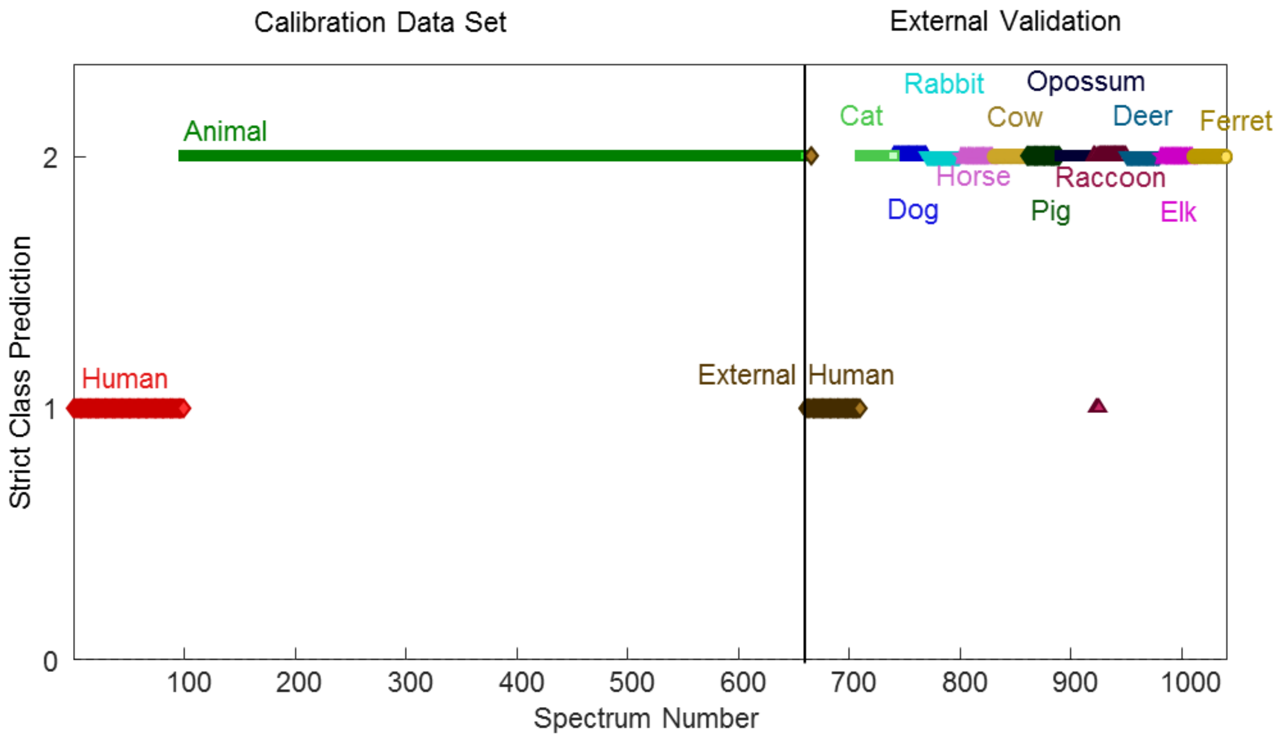

Supplementary Fig. 2 Strict class prediction results by the human–animal PLSDA model with five latent variables for blood samples. Human blood spectra (red diamonds) are assigned a value of 1 and animal blood spectra (green squares) are assigned a value of 2. All spectra of external samples are on the right side of the plot (spectra numbers 661–1040).

Supplementary Table 2. Prediction results of external samples by the human–animal PLSDA model using five latent variables. The table shows results at the spectral level and the donor level (when more than 50% of spectra from one donor are correctly classified).

	**Predicted as human (spectral level)**	**Predicted as animal (spectral level)**	**Donor-level prediction**
**Human**	49	1	5/5
**Cat**	0	30	3/3
**Dog**	0	30	3/3
**Rabbit**	0	30	3/3
**Horse**	0	30	3/3
**Cow**	0	30	3/3
**Pig**	0	30	3/3
**Opossum**	0	30	3/3
**Raccoon**	3	27	3/3
**Deer**	0	30	3/3
**Elk**	0	30	3/3
**Ferret**	0	30	3/3
