# Supplementary material for: Discrimination between human and animal blood by attenuated total reflection Fourier transform-infrared spectroscopy
Source: Commun Chem. 2020 Dec 10;3:178. doi: 10.1038/s42004-020-00424-8 (PMC9814708; doi:10.1038/s42004-020-00424-8)
Supplement: Supplementary file 2 — Reporting Summary [file 42004_2020_424_MOESM2_ESM.pdf]

nature research

Corresponding author(s): Igor K. Lednev

Last updated by author(s): Sep 8, 2020

Reporting Summary

Nature Research wishes to improve the reproducibility of the work that we publish. This form provides structure for consistency and transparency in reporting. For further information on Nature Research policies, see our Editorial Policies and the Editorial Policy Checklist.

Statistics

For all statistical analyses, confirm that the following items are present in the figure legend, table legend, main text, or Methods section.

n/a	Confirmed	
[ ]	[x]	The exact sample size (n) for each experimental group/condition, given as a discrete number and unit of measurement
[ ]	[x]	A statement on whether measurements were taken from distinct samples or whether the same sample was measured repeatedly
[x]	[ ]	The statistical test(s) used AND whether they are one- or two-sided<br>*Only common tests should be described solely by name; describe more complex techniques in the Methods section.*
[x]	[ ]	A description of all covariates tested
[ ]	[x]	A description of any assumptions or corrections, such as tests of normality and adjustment for multiple comparisons
[x]	[ ]	A full description of the statistical parameters including central tendency (e.g. means) or other basic estimates (e.g. regression coefficient) AND variation (e.g. standard deviation) or associated estimates of uncertainty (e.g. confidence intervals)
[x]	[ ]	For null hypothesis testing, the test statistic (e.g. F, t, r) with confidence intervals, effect sizes, degrees of freedom and P value noted<br>*Give P values as exact values whenever suitable.*
[x]	[ ]	For Bayesian analysis, information on the choice of priors and Markov chain Monte Carlo settings
[x]	[ ]	For hierarchical and complex designs, identification of the appropriate level for tests and full reporting of outcomes
[x]	[ ]	Estimates of effect sizes (e.g. Cohen's d, Pearson's r), indicating how they were calculated

Our web collection on statistics for biologists contains articles on many of the points above.

Software and code

Policy information about availability of computer code

Data collection	PerkinElmer, Inc. Spectrum software version 6.0.2.0025 (PerkinElmer, Inc., Waltham, MA)
Data analysis	PLS Toolbox 8.5.2 (Eigenvector Research, Inc., Wenatchee, WA) operating in Matlab R2017b version 9.3.0.713579 (MathWorks, Inc., Natick, MA)

For manuscripts utilizing custom algorithms or software that are central to the research but not yet described in published literature, software must be made available to editors and reviewers. We strongly encourage code deposition in a community repository (e.g. GitHub). See the Nature Research guidelines for submitting code & software for further information.

Data

Policy information about availability of data

All manuscripts must include a data availability statement. This statement should provide the following information, where applicable:

- Accession codes, unique identifiers, or web links for publicly available datasets
- A list of figures that have associated raw data
- A description of any restrictions on data availability

The data sets generated during and/or analyzed during the current study are available from the corresponding author on reasonable request.

Field-specific reporting

Life sciences study design

All studies must disclose on these points even when the disclosure is negative.

Sample size	104 samples: 15 human blood samples and 89 animal blood samples. The data set was split into calibration (training) set and validation set. The validation data set contained a minimum of 30% of the samples from each class: five human samples and three samples from each animal group. Additional validation was performed using external species (deer, elk, and ferret) from outside the training data set.
Data exclusions	The spectral ranges of 4000–2800 and 1800–600 cm−1 were used as they showed contributions in the biochemical compositions of the biological samples. [25,26]<br>[25] Orphanou, C.-M., Walton-Williams, L., Mountain, H. & Cassella, J. The detection and discrimination of human body fluids using ATR FT-IR spectroscopy. Forensic Sci. Int. 252, e10-e16, doi:http://dx.doi.org/10.1016/j.forsciint.2015.04.020 (2015).<br>[26] Staniszewska, E., Malek, K. & Baranska, M. Rapid approach to analyze biochemical variation in rat organs by ATR FTIR spectroscopy. Spectrochim. Acta A 118, 981-986, doi:https://doi.org/10.1016/j.saa.2013.09.131 (2014).
Replication	*Describe the measures taken to verify the reproducibility of the experimental findings. If all attempts at replication were successful, confirm this OR if there are any findings that were not replicated or cannot be reproduced, note this and describe why.*
Randomization	The samples for slit of 70/30 for calibration and validation data set were chosen randomly. Moreover, the models were validated using internal cross-validation (CV). The CV process divides the data set so that some subsets are used for training the model and some are used for validation. The procedure is continued until all spectra are used for validation.
Blinding	Blinding investigators was not relevant to this study since statistical models were used in order to perform classification. External validation was used with samples from outside of the training data set and their identity was unknown to the statistical models constructed.

Reporting for specific materials, systems and methods

We require information from authors about some types of materials, experimental systems and methods used in many studies. Here, indicate whether each material, system or method listed is relevant to your study. If you are not sure if a list item applies to your research, read the appropriate section before selecting a response.

Materials & experimental systems

n/a	Involved in the study	
☒	☐	Antibodies
☒	☐	Eukaryotic cell lines
☒	☐	Palaeontology and archaeology
☒	☐	Animals and other organisms
☒	☐	Human research participants
☒	☐	Clinical data
☒	☐	Dual use research of concern

Methods

n/a	Involved in the study	
☒	☐	ChIP-seq
☒	☐	Flow cytometry
☒	☐	MRI-based neuroimaging
